# Molecular Mechanisms of New Bone Formation in Axial Spondyloarthritis

**DOI:** 10.31138/mjr.33.1.115

**Published:** 2022-04-15

**Authors:** Kalliopi Klavdianou, Anastasia Kanellou, Dimitrios Daoussis

**Affiliations:** 1 Department of Rheumatology, “Asklepieion” General Hospital, Athens, Greece,; 2 Department of Anatomy-Histology-Embryology, Laboratory of Bone and Soft Tissue Studies, University of Patras Medical School, Patras, Greece,; 3 Department of Rheumatology, Patras University Hospital, University of Patras Medical School, Patras, Greece

**Keywords:** axial spondyloarthritis, ankylosing spondylitis, new bone formation, osteoblastogenesis, Wnt, Hedgehog, IL-17, TNF, serotonin

## Abstract

Axial spondyloarthritis (axSpA) is a disease characterised by new bone formation. Biologic agents targeting TNFα or IL-17 are used widely and are very effective in controlling symptoms and improving quality of life in these patients. However, the effect of biologics on radiographic progression is still not entirely known. The most crucial question to be addressed is whether new bone formation in the context of axSpA is linked to the inflammatory process. If new bone formation and inflammation are interconnected, then long-term suppression of inflammation with biologic agents may eventually lead to inhibition of ankylosis. On the other hand, if these processes are totally uncoupled then biologics may not have an obvious effect on radiographic progression. In this case, targeting pathways that control new bone formation may be a more feasible approach to retard radiographic progression in axSpA. The molecular mechanisms involved in new bone formation in axSpA have been extensively investigated throughout the last years. In this narrative review we summarise the data regarding the mechanisms of new bone formation in axSpA.

## INTRODUCTION

Spondyloarthritides (SpA) are a heterogeneous group of rheumatic diseases that share many features. They have a common genetic background and are characterised by a tendency for inflammation at sites exposed to stress either mechanical, such as the enthesis or microbial, such as the gut and skin. SpA are divided into two major categories: axial and peripheral, based on whether the main manifestations involve the axial or the peripheral skeleton. Axial spondyloarthritis (axSpA) is typically characterised by ectopic new bone formation that eventually leads to decreased range of motion and spinal ankylosis.^1^ Bone metabolism in axSpA is an extremely complex process. At sites of increased mechanical stress there is a tendency towards new bone formation, which produces characteristic syndesmophytes. On the other hand, these patients may overall have decreased bone mass potentially due to the effect of the systemic inflammatory process. The treatment of axSpA has been revolutionized with the introduction of biologic agents that target TNFα or IL-17; these cytokines are crucially involved in the pathophysiologic process of the disease.^2^ These agents have shown a remarkable clinical efficacy, since they improve pain, physical function and quality of life. However, it is still debatable whether biologics can inhibit or at least retard radiologic progression and ankylosis. In the short term, biologics have been shown to have no effect on the process of new bone formation.^3,4^ However, evidence accumulating during the last years, points to the direction that long-term anti-TNFα treatment may slow down the rate of radiographic progression.^5^ The most critical question to be addressed is whether new bone formation in the context of axSpA is linked to the inflammatory process. If ectopic bone formation and inflammation are interconnected, then long-term suppression of inflammation with biologic agents may eventually lead to inhibition of ankylosis. On the other hand, if these processes are totally uncoupled, then biologics may not have an obvious effect on radiographic progression. In this case, targeting pathways that control new bone formation may be a more feasible approach to retard radiographic progression in axSpA even though this strategy may potentially affect overall bone mass and lead to osteoporosis. The molecular mechanisms involved in new bone formation in axSpA have been extensively investigated throughout the last years. In this narrative review we summarise the data regarding the mechanisms of new bone formation in axSpA.

## WNT PATHWAY

### How the Wnt pathway works

**Figure 1:**
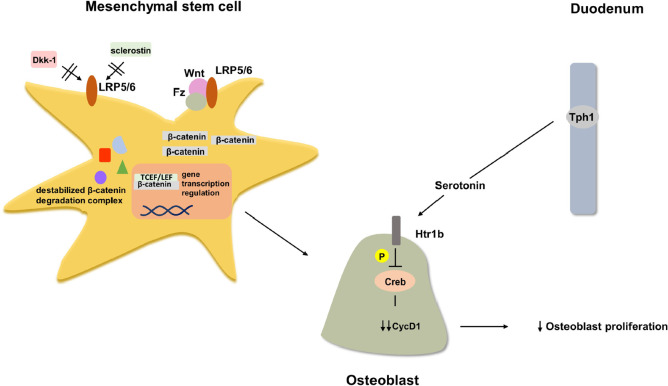
**Wnt signalling pathway and serotonin in bone formation.** A Wnt ligand binds to Frizzled (Fz)/LRP5/6 coreceptor complex and β-catenin degradation complex is destabilized. β-catenin accumulates to cytoplasm and enters the nucleus. In the nucleus, β-catenin along with TCF/LEF regulates the transcription of Wnt target genes leading to differentiation of mesenchymal cells to osteoblasts**.** Dkk-1 and sclerostin inhibit the pathway. Peripheral serotonin is produced in the duodenum by Tph1. Free serum serotonin binds to its receptor Htr1b on osteoblasts. The binding leads to decreased CREB phosphorylation and subsequently to reduced transcription of cell proliferation genes such as Cyclin D1(CycD1), resulting in decreased osteoblast proliferation.

The main role of developmental pathways is organizing growth and development during embryogenesis. However, they can also remain active during adulthood and mediate homeostatic functions in response to tissue injury.^6^ Wnt is an embryonic pathway implicated in growth, homeostasis and bone formation.^7,8^ The best described Wnt signalling cascade is the so called ‘canonical’ pathway. The ligands of the pathway are 19 Wnt proteins that bind to a complex consisting of Frizzled transmembrane receptors and the LRP5/6 (low-density lipoprotein receptor-related protein) coreceptor. The main pathway mediator is β-catenin. In absence of a Wnt ligand, cytoplasmic β-catenin is phosphorylated by a protein complex and subsequently driven to proteosomic degradation. The binding of a Wnt ligand to the Frizzled/LPR5/6 coreceptor, destabilizes the degradation complex and β-catenin translocates to the nucleus, where it acts as a transcriptional coactivator with TCF/LEF(T-cell factor/lymphoid enhancer factor) to activate Wnt target genes.^9,10^ The pathway is extracellularly regulated by several inhibitors such as Dickkopf-1 (Dkk-1) and sclerostin.^11–13^ These are diagrammatically depicted in **Figure 1**. Gain or loss of function mutations of LRP5 gene leading to high or low bone density, respectively, provided the first evidence linking the Wnt pathway to osteoblastogenesis.^14,15^ At the late stages of endochondral bone formation Wnt has been shown to stimulate the production of hypertrophic chondrocytes, which are finally replaced by bone.^16^

### In vitro data and animal models linking the Wnt pathway to new bone formation

Taking into account the strong link between the Wnt pathway and osteoblastogenesis it was no surprise that it was investigated in the context of axSpA, a disease characterized by new bone formation. Diarra et al. explored mechanisms inducing bone loss in murine inflammatory arthritis and found that administration of anti-Dkk1 monoclonal antibody (mAb) resulted in protection against erosions as well as to new bone formation without affecting inflammation.^17^ This finding indicated that these two processes are uncoupled. The authors were the first to describe that Dkk-1 regulates the balance between bone formation and bone loss, with low expression leading to bone formation and high expression leading to bone loss. They showed that bone formation is suppressed by TNFα induced Dkk-1 upregulation and that *in vitro* inhibition of Dkk-1 with a mAb induces bone formation. On the other hand, Li et al. suggested that low grade inflammation has a role in bone formation.^18^ They created an *in vitro* cell system mimicking the microenvironment of sites of new bone formation. Treatment of progenitor bone cells with low dose TNFα, induced the expression of Wnt proteins and new bone formation, while treatment with high-dose TNFα induced expression of Dkk-1. These findings suggested an inflammation intensity-dependent expression of Wnt proteins, indicating that at least *in vitro* Wnt could be a link between inflammation and new bone formation in SpA. Further experiments revealed that both canonical and non-canonical Wnt pathways are necessary for new bone formation. Blocking canonical or non-canonical Wnt pathway in two mouse models of ax-SpA, reduced new bone formation but best results were obtained by simultaneous blocking of both pathways. However, the authors could not identify a correlation between inflammation intensity and new bone formation in these models.

Uderhadt et al. treated a transgenic TNF mouse that displays erosive sacroiliitis with anti-Dkk1 mAb.^19^ The subsequent enhancement of Wnt signalling led to new bone formation and ankylosis, providing evidence that Wnt has a role in new bone formation in the axial skeleton. To substantiate this, SOST (the gene encoding sclerostin) and DKK1 gene expression levels were found to be downregulated in the spine of a proteoglycan-induced spondylitis (PGISp) mouse model.^20^ However, treatment of PGISp mice with recombinant sclerostin failed to reduce osteoproliferation in the axial skeleton.^21^ In another study the authors analysed the effect of Wnt5a on cultured murine tendon cells, chondrocytes and osteoblasts. They found that the addition of Wnt5a decreased gene expression of differentiation markers and mineralization in cultured chondrocytes and reduced alkaline phosphatase activity at entheses. On the contrary, Wnt5a, stimulated the expression of ossification markers in cultured osteoblasts and increased the bone volume of the tibial plateau of the cultured explants, providing evidence of opposing effects of Wnt signalling observed in enthesis/chondrocytes and osteoblasts/bone.^22^

Several studies have investigated the role of epigenetic contribution in AS. microRNAs (MiRNAs) are small, noncoding RNA molecules post-transcriptionally regulating the expression of various genes.^23^ miRNAs seem to interact with signalling molecules during osteoblast differentiation. miRNA29a has been shown to stimulate bone formation by modulating Wnt signalling,^24^ possibly by downregulating Dkk-1 expression.^25^ miRNA-96 is increased in a SpA mouse model and has been shown to promote osteoblast differentiation and bone formation through downregulation of sclerostin gene (SOST) in osteoblasts.^26^

The above data provide evidence in favour of a strong link between the Wnt pathway and the process of new bone formation and therefore justify in depth analysis of this pathway in the context of axSpA.

### Humans

The role of the Wnt pathway in bone formation has been assessed in human SpA especially in patients with ankylosing spondylitis (AS). Most studies focus on Wnt pathway inhibitors Dkk-1 and sclerostin by analysing serum levels, tissue expression and function.

#### Dkk-1

In a study of our department we found that circulating Dkk-1 levels are higher in patient with AS but the functional levels of the molecule, assessed with a functional ELISA are decreased, indicating that this molecule might be dysfunctional in AS.^27^ In further mechanistic experiments, we cultured Jurkat cells and treated them with serum from AS patients or healthy controls. The addition of AS serum resulted in higher Wnt activation compared with that seen by the addition of serum from healthy subjects, suggesting that Dkk-1 is dysfunctional in AS and may contribute to new bone formation. The high circulating Dkk-1 levels in early axSpA has been verified in the large DESIR cohort.^28^ Disease duration was shown to be of importance for circulating Dkk-1 levels with significantly higher levels in naïve patients with early ax-SpA compared with patients with established axSpA.^29^ Finally, a meta-analysis of seven studies including 300 AS patients confirmed that circulating Dkk-1 levels are higher in AS compared to healthy subjects.^30^ These data indicate that patients with AS have high circulating but low functional Dkk-1 levels with ex vivo assays suggesting a compromised function.

The next critical question to be addressed is the expression of this molecule at tissues of pathophysiologic relevance for axSpA but only limited data are available. DKK1 gene and protein levels were downregulated in hip synovial tissues of AS patients compared to healthy subjects.^31^ Primary fibroblasts from AS hip capsules were cultured and exhibited an increased growth rate, excessive proliferation, and a decreased apoptotic rate. Flow cytometric analysis showed that downregulation of DKK1 by shRNA (short hairpin RNA) silencing led to an increase in the proliferation of AS fibroblasts and increase of β-catenin, while upregulation had the opposite effects, indicating that DKK1 is involved in the proliferation of AS fibroblasts through Wnt signalling.

Finally, genetic studies have provided some useful insights. In a French cohort of patients with early axSpA (DESIR), no association between DKK1 SNPs (single nucleotide polymorphisms), Dkk-1 circulating levels and structural damage was found, suggesting that in axS-pA Dkk-1 circulating levels may not be genetically determined.^28^ Another study exploring the presence of genetic associations with radiographic severity in AS did not detect association with DKK1 gene SNPs.^32^ The two latter studies point to the direction that Dkk1 dysfunction in AS is not genetically determined but possibly caused by posttranscriptional changes.

#### Sclerostin

Osteocytes in the periarticular bone of patients with AS have significantly reduced expression of sclerostin as compared with that of osteocytes along normal joints.^33^ whereas serum sclerostin levels were found decreased in patients with AS compared to healthy subjects.^34^ Serum anti-sclerostin autoantibodies have been detected in healthy individuals but at significantly higher levels in AS patients.^35^ However, it is not known if these autoAbs have functional effects and contribute to new bone formation in AS.

### Dkk-1 and sclerostin as biomarkers

Several researchers have addressed the question of whether Wnt antagonists could serve as biomarkers of bone density and radiographic progression in AS. Serum functional^36^ and circulating^37^ Dkk-1 levels have been found to be inversely correlated with radiographic severity of AS. More specifically, high functional Dkk-1 serum levels were shown to protect from syndesmophyte formation^38^; on the other hand, low serum sclerostin levels in patients with AS were associated with the formation of new syndesmophytes.^33^ Rademacher et al. explored if adding biomarkers to clinical parameters could improve prediction of radiographic spinal progression in axSpA.^39^ After testing combined clinical parameters and biomarkers (among them sclerostin) in the models, they concluded that the addition of biomarkers improved predictive value for radiographic spinal progression in axSpA, but the added value was small.

The above data indicate that both Dkk-1 and sclerostin may be used as biomarkers in axSpA. However, relevant evidence is not powerful and therefore these molecules are not yet ready for use in everyday clinical practice.

### Potential links between cytokines and the Wnt pathway

Inflammatory cytokines such as TNFα, IL-6 and IL-17 may regulate both osteoclastogenesis and osteoblastogenesis directly or indirectly. A critical question is whether these cytokines may interact with the Wnt pathway in axSpA and if that interaction could possibly have an effect on bone formation. There are several experimental data that provide insights regarding potential interactions between IL-6, IL-17, TNFα and the Wnt pathway.

Yeremenko et al found that local Dkk-1 levels in the inflamed synovia of patients with peripheral SpA were similar to those in the inflamed joints of patients with RA and that in contrast with TNF, IL-6 has a suppressive effect on Dkk-1 production.^40^

It has been reported that that IL-17A can suppress DKK1 mRNA levels in human mesenchymal stem cells.^41^ In a recent study it was found that treatment of human periosteum derived cells (hPDCs) with IL-17A and IL-17F induced bone formation *in vitro*. Similar effects were seen when treating hPDCs with Th17, γδ-T cell supernatants or serum from patients with AS. Notably, dual inhibition of IL-17A and IL-17F in human hPDCs treated with T-cell supernatants or serum from patients with AS led to inhibition of bone formation. IL-17 neutralisation resulted in enhanced DKK1 expression in hPDC cultures compared to those exposed to T-cell supernatants alone, indicating that the osteogenesis inhibition mediated by IL-17 blockade could at least partially be attributed to Wnt inhibition.^42^

Another study explored the effects of TNF blockade on SKG mice, a model which develops the clinical characteristics of SpA including sacroiliac joint involvement, vertebral inflammation, enthesitis, peripheral arthritis, uveitis, and bowel inflammation following curdlan administration.^43^ The authors treated the mouse with a TNF blocker and found that spinal inflammation assessed with 18F-FDG uptake in PET-MRI and inflammatory cells infiltration in spinal tissue was decreased.^44^ Accumulation of hydroxyapatite indicating osteoblast activity was assessed using a fluorescent in vivo bisphosphonate imaging agent before and following treatment. TNF inhibition did not reduce osteoblastic activity assessed by imaging and did not restore Dkk1 and sclerostin serum levels suggesting the presence of other TNF independent osteoproliferation pathways.

## HEDGEHOG PATHWAY

Another developmental pathway that has been implicated in the process of new bone formation is the Hedgehog (HH) pathway. In axSpA, syndesmophytes develop mainly through endochondral ossification and evidence indicates that the HH pathway is crucially involved in this process.^45,46^ This pathway consists of 3 ligands (Indian, Sonic and Desert hedgehog) that bind the receptor Patched-1; finally, an intracellular signal is transduced by mobilizing the group of transcription factors named glioma associated oncogene homologs (Gli). Indian hedgehog (Ihh) is produced by prehypertrophic chondrocytes and is the main driver of endochondral ossification.^47,48^ The HH pathway has been linked to osteophyte formation in the context of osteoarthritis (OA). In an experimental model of OA, induced by surgical methods, mice that were genetically modified to display increased HH signaling developed more pronounced osteoarthritic changes compared to WT mice. Of note, therapeutic targeting of the HH pathway in this animal model led to attenuation of OA severity and osteophyte formation providing strong evidence that the HH pathway is a master regulator of new bone formation in the context of degenerative diseases. Humans with OA also appear to exhibit enhanced HH pathway activation.^49^

Further evidence linking the HH pathway to new bone formation comes from an experimental model of inflammatory arthritis. The investigators used a HH pathway inhibitor and found that treated mice developed less osteophytes compared to untreated controls. Notably, HH pathway blockade did not have any effect on inflammation; inhibition of osteophyte formation was achieved by specifically blocking the endochondral ossification process.^50^ These data indicate that HH pathway inhibitors could be a therapeutic tool to attenuate new bone formation in the context of inflammatory arthritis.

The most convincing data pointing to the direction of a tight link between spinal new bone formation and the HH pathway comes from a mouse model that was genetically modify to display increased HH pathway activity at spinal chondrocytes.^51^ Interestingly, these mice developed fusion of their spine without inflammation indicating that in this experimental setting inflammation and new bone formation are two completely uncoupled processes.

In a study performed at our Department we found that patients with AS not receiving biologics had increased Ihh levels compared to patients with RA not receiving biologics and healthy subjects.^52^ This finding may have pathogenetic implications; increased Ihh levels may enhance HH signalling and potentially boost the process of endochondral ossification/new bone formation in patients with AS. Moreover, we found that TNF blockers decrease Ihh levels in patients with AS. In further functional experiments we evaluated the effect of serum derived from patients with AS prior to and following anti-TNF treatment, on the expression of HH pathway target genes by using an osteoblast-like cell line model. We found a significant downregulation of HH pathway activity following treatment with TNF blockers. These data suggest that anti-TNF treatment might affect Ihh levels and potentially HH pathway activation.

The experimental evidence presented above pinpoint the critical role of the HH pathway in the process of endochondral ossification and new bone formation in the context of degenerative or inflammatory diseases. Of note, HH pathway inhibitors are clinically available and have been used in cancer exhibiting an acceptable safety profile.

## SEROTONIN

Serotonin is a bioamine produced in the central nervous system (CNS) by the enzyme tryptophane hydroxylase 2 (Tph2) and in enterochromaffin cells of the duodenum by tryptophane hydroxylase 1 (Tph1).^53,54^ It does not cross the blood brain barrier and brain derived serotonin (BDS) has different actions than gut derived serotonin (GDS), the latter accounting for 95% of the total. In the circulation serotonin is taken up and stored by platelets but a small amount, approximately 5%, remains free. Free serotonin is believed to have endocrine actions. Serotonin receptors have been identified in many cell types including osteoblasts.^55–57^ Experimental and clinical data indicate that serotonin has a role in regulation of osteoblastogenesis with BDS enhancing bone formation and decreasing bone resorption and GDS acting as a brake to osteoproliferation.^58^

Yadav et al suggested that GDS binds to its receptor Htr1b (hydroxytryptamine receptor 1b) on osteoblasts leading to decreased CREB (cAMP response element-binding protein) phosphorylation, subsequent decrease of transcription of proliferation genes and finally to downregulation of osteoblast proliferation.59 An illustration of how serotonin may inhibit osteoblast proliferation is presented in **Figure 1**.

The same group inhibited Tph1 in mice by treating them with LP533401.^60^ This molecule, designed for use in irritable bowel syndrome, inhibits GDS without affecting BDS.^61^ Pharmacological inhibition of Tph1 decreased the levels of GDS in these mice, prevented bone loss and could treat osteoporosis.^60^ *In vitro*, GDS significantly reduces osteogenic differentiation and mineralization of rat calvarial cells with concomitant reduction of osteoblast marker genes.^62^ These findings indicate the existence of a gut/bone axis.^63,64^

Clinical data for bone regulation through GDS are available. Children with autism with increased serum serotonin levels^65^ present with significant decrease of cortical thickness.^66^ Serum serotonin levels of postmenopausal women with osteoporosis are inversely correlated with total body and spine bone mineral density.^67^ At the same time serum serotonin levels in patients with high bone mass syndrome associated with gain of function mutations of LRP5 were measured lower than healthy controls, supporting the hypothesis that serotonin levels mediate increased bone mass in these patients.^68^ An inverse correlation of serotonin levels with BMD was identified in these patients.^69^

Selective serotonin reuptake inhibitors (SSRIs), widely used for depression and other psychiatric disorders have been correlated with bone loss and increased fracture risk.^70,71^ These agents could affect serotonin at many levels including CNS, platelets and skeletal microenvironment. A possible explanation for bone loss in patients on SSRIs is that local concentration of serotonin is increased in skeletal microenvironment due to reuptake inhibition by osteoblasts, resulting in longer exposure of osteoblasts to serotonin.

In a study of our Department we found that patients with AS have lower serum serotonin levels than patients with RA and healthy subjects.^72^ Treatment with TNF blockers associates with even lower serotonin levels in AS patients. In an effort to explore whether low serotonin levels in AS has functional effects we treated a human osteoblast-like cell line with AS sera and found increased CREB phosphorylation indicating serotonin signalling attenuation. In an effort to explore why patients with AS have low serum serotonin, we found that Tph1 gene expression was suppressed in duodenum of patients with inflammatory bowel disease compared to healthy subjects. Taking into account that most patients with AS have subclinical gut inflammation we may hypothesize that Tph1 may be similarly under expressed in AS gut. These data suggest that GDS could at least partially control bone formation in patients with axSpA.

The data presented suggest a role of serotonin in regulation of bone formation, but further studies are required to determine if we can modulate peripheral serotonin therapeutically to treat diseases with low or high bone formation.

## IL-17

During the last decade, accumulating evidence points to the direction that the IL-23/IL-17 axis is crucially involved in the pathogenesis of SpA. The concept that IL-23 may stimulate cells residing at the entheses such as innate-like lymphoid cells type 3 or γδ T cells to produce IL-17 has revolutionized our understanding of SpA pathophysiology. However, the most convincing data regarding the critical role of IL-17 come from clinical studies indicating the clinical efficacy of IL-17 inhibitors in axSpA. IL-17 inhibitors are approved for the treatment of axSpA since they are able to reduce pain and functional impairment and increase quality of life in patients with the disease. The role of IL-17 in the process of bone remodelling is not well understood. Nevertheless, recent evidence shows that IL-17 is implicated in mechanisms of new bone formation.

Robust experimental evidence indicate that IL-17 is a strong inducer of osteoclastogenesis.^73–76^ In mouse models of arthritis, IL-17 appears to cause bone loss by promoting osteoclast differentiation indirectly, through the stimulation of RANKL (receptor activator of NF-kappa B ligand) expression by osteoblasts.^77^ RANKL is a molecule expressed mainly by cells of mesenchymal lineage and induces osteoclast differentiation by binding to the RANK receptor found on osteoclast precursor cells. Other data show that IL- 17 may influence osteoclastic function directly as well, increasing RANK expression at osteoclast precursors and therefore their response to RANKL stimulation.^78^ Additionally, IL-17 may activate the production of other pro-inflammatory cytokines which are known to affect osteoclastogenesis such as TNF, IL-1 and IL-6.^79^

On the other hand, studies about the effect of IL- 17 on osteoblasts, cells that produce bone matrix, exhibit rather conflicting results. In vitro studies with primary calvarial osteoblasts from rodents indicated that IL-17 inhibited osteoblast differentiation through the inactivation of the WNT pathway.^80–82^ Also, IL-17 deficient mice developed more periosteal bone than wild type animals in an induced arthritis model.^82^ Furthermore, overexpression of IL-17 from the skin, leads to systemic bone loss due to osteoblast inactivation through WNT pathway inhibition in psoriasis mouse models. The bone loss phenotype was reversed with IL-17 blockade, enhancing the notion that IL-17 causes bone loss.^83^ In support of this concept, IL-17 blockade and IL-17RA deficiency protected ovariectomized mice from bone loss.^84,85^ The above results indicate that IL-17 suppresses osteoblasts.

On the other hand, IL-17 induced the proliferation and osteoblastic differentiation of mesenchymal stromal cells (MSCs) cultured *in vitro*.^86,87^ The osteogenic effect seems to be more intense when IL-17 acts synergistically with TNF-a or BMP2.^41,88^ Notably, Ono et al. demonstrated that IL-17 is crucial for fracture healing in a drill hole fracture model, as it seems to promote the differentiation and function of osteoblasts.^89^ Also, in another ovariectomy model, bone loss was more prominent in mice deficient for IL-17RA.^90^ These results indicate that IL-17 promotes osteoblastic activity and thus bone formation and appear to contradict the previously reported data showing an inhibitory effect on osteoblasts. However, it seems that the effect of IL-17 depends on the cell type and the stage of differentiation of the cells used in each study. For example, treatment of undifferentiated mesenchymal stem cells (MSCs) with IL-17 induces cell proliferation and osteoblast differentiation. In sharp contrast, in already committed calvarial osteoblasts, IL-17 suppresses further differentiation and function. Moreover, the dosage, the time point, the duration of exposure and the disease model used can probably influence the effect of IL-17 on osteoblast function. Osta et al. demonstrated that the cellular environment plays an important role in the osteogenic effect of IL-17. IL-17 induced osteoblast differentiation and function of fibroblast-like synoviocytes derived from osteoarthritis or rheumatoid arthritis patients, while it caused bone erosion on bone explants cultured ex vivo. This implies that the net effect of 1L-17 on bone metabolism may depend on the balance between osteoblast and osteoclast function.^91^

Regarding AS, recent preclinical studies show that IL-17 may play a key role in abnormal bone formation. IL-17 promotes osteoblast differentiation of mesenchymal lineage cells derived from bone and synovial tissues of AS patients^92,93^ and boosts osteoblastic activity via JAK2/STAT3 signalling.^92^ Anti-IL-17 antibody attenuated the osteogenic differentiation and function of bone derived cells from AS patients and healthy individuals cultured in the presence of AS sera.^92^ Additionally, blockade of IL-17 in HLA-B27-transgenic rat models of AS resulted in delayed manifestation of arthritis and spondylitis, reduced arthritis severity and diminished bone formation.^93^ Of note, in a recent study it has been shown *in vitro* that IL-17 bound to neutrophil extracellular traps induces osteoblast differentiation of human mesenchymal stem cells more effectively that soluble IL-17 adding further evidence in favour of a critical role of this cytokine in the bone forming process.^94^

**Figure 2:**
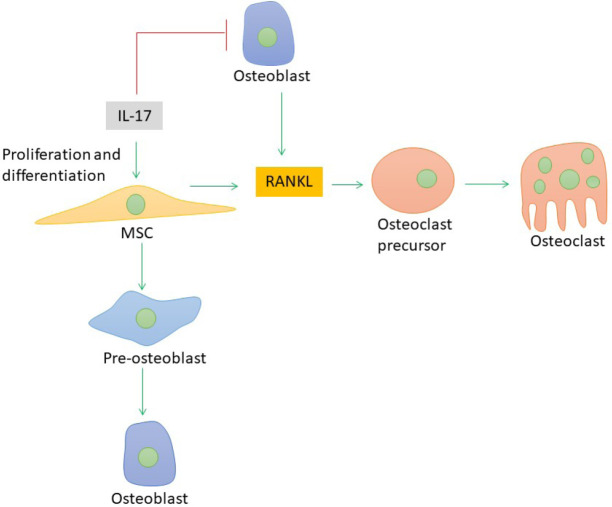
The effect of IL-17 on osteoblast and osteoclast differentiation. IL-17 induces the proliferation and osteoblastic differentiation of MSCs. On the other hand, IL-17 inhibits further differentiation of committed osteoblasts. Also, IL-17 increases the production of RANKL from MSCs and osteoblasts promoting osteoclast differentiation.

To conclude, current data suggest that IL-17 is a key molecule for the pathogenesis of AS. Not only it is crucial for the inflammatory process but also may be directly implicated in the process of new bone formation. IL-17 seems to be involved both in bone loss and abnormal bone formation in distinct anatomical sites in AS patients. The potential role of IL-17 in regulating new bone formation is shown in **Figure 2**.

## MECHANICAL STRESS

The critical role of mechanical stress as a trigger or driver of new bone formation cannot be overlooked. The target tissue in SpA is the enthesis which is the part of the human body that receives enormous amounts of mechanical load and is prone to repetitive microtrauma.^95^ In an experimental model (collagen antibody induced arthritis in DBA/1 mice), it was shown that decreasing the mechanical load on hind paws by tail suspension led to attenuation of osteophyte formation compared to mice kept in standard cages.^96^ These data highlight the role of mechanical strain as a driver/enhancer of new bone formation. Further mechanistic studies provided evidence that in animal models, inflammation is more likely to occur and become chronic at sites exposed to mechanical stress and that this process is mostly controlled by stromal cells and not lymphocytes.^97,98^ The above data suggest that mechanical strain is a crucial regulator of inflammatory responses and new bone formation in specific experimental settings. The role of mechanical strain in new bone formation is also underscored by the fact that most animal models of SpA do not display the typical features of the human counterpart such as sacroiliitis or spinal ankylosis. The most plausible explanation for this is the fact that mice are quadrupedal and therefore the pelvis and spine do not receive major mechanical loads; this may explain why these sites are protected from new bone formation in mice. In sharp contrast, humans are bipedal, and thus the axial skeleton is subjected to intense mechanical strain that may lead to new bone formation. The role of mechanical stress in humans with axial SpA in also shown by clinical data indicating that AS patients with “white collar” jobs have less bone formation compared to manual workers.^99^

Based on these, we may conclude that mechanical strain may facilitate the process of new bone formation. Therefore, patients with axSpA should probably avoid exercises that transduce excessive mechanical loads to the axial skeleton; this may potentially lead to reduced risk for radiographic progression.

## CONCLUSIONS

Most data indicate that new bone formation in the context of SpA is mostly performed through endochondral ossification. Evidence suggests that this process is initiated by mesenchymal stem cells that are recruited and differentiate into osteoblasts, under the control of developmental pathways. This is why developmental pathways such as the Wnt and HH pathways have received considerable attention as key drivers of ectopic bone formation in the context of SpA. A critical question is whether we may target developmental pathways therapeutically in order to inhibit new bone formation in axSpA taking into account the key functions of these pathways. Several data indicate that this approach might be feasible. Romosozumab, an inhibitor of sclerostin, is already used as an anabolic treatment in osteoporosis with an acceptable tolerability profile.^100^ Studies on molecules targeting Dkk-1 in various malignancies are in early stages.^101,102^

Up until now, the only available targeted therapies in axSpA are biologics targeting TNF or IL-17. These drugs exhibit a strong anti-inflammatory action and effectively reduce symptoms in patients with axSpA. Evidence accumulating during the last years indicate that TNF blockade may retard radiographic progression,^103^ especially if administered early on or following long-term therapy.^104^ In specific animal models of SpA, evidence indicate that blocking IL-17 may lead to attenuation of osteophyte formation but this was not a consisted finding in all studies. The long-term effect of IL-17 inhibition on radiographic progression is not known but in the midterm it appears that IL-17 blockers associate with a relatively slow risk of progression.^105^ A clinical study directly comparing the effects of TNF vs IL-17 blockers on clinical parameters and new bone formation in patients with axSpA is currently running and results are eagerly awaited.

If indeed biologics are shown to inhibit radiographic progression the next question is how they mediate this effect. Some data indicate that this could be achieved through inhibition of inflammation. However, based on data presented in this review yet another plausible explanation should be considered. IL-17 and TNF may directly or indirectly interact with developmental pathways; therefore, TNF or IL-17 blockers may modulate the activity of these pathways and potentially the process of new bone formation.

Mechanical strain also appears as an important mediator of new bone formation and should not be overlooked. Typical AS is more prominent in males which are usually subjected to more mechanical stress. Furthermore, physically demanding jobs have been associated with more ectopic bone formation in patients with axSpA. These clinical data are reinforced by strong experimental data pointing to the direction that new bone formation is driven by mechanical strain.

For now, the only feasible approaches to potentially reduce the likelihood of radiographic progression in patients with AxSpA appears to be the control of inflammation with the early introduction of a biologic if necessary, the avoidance of excessive mechanical stress in axial skeleton, and, perhaps, smoking cessation. In the future, we may be able to directly target specific pathways that control osteoblastogenesis. Even though a significant progress has been made towards a better understanding of the mechanisms controlling new bone formation in axSpA, we still have a long way to go.
